# Effect of dietary betaine supplementation on the liver transcriptome profile in broiler chickens under heat stress conditions

**DOI:** 10.5713/ab.23.0228

**Published:** 2023-08-30

**Authors:** Deok Yun Kim, Gi Ppeum Han, Chiwoong Lim, Jun-Mo Kim, Dong Yong Kil

**Affiliations:** 1 Department of Animal Science and Technology, Chung-Ang University, Anseong 17546, Korea

**Keywords:** Betaine, Broiler Chicken, Growth Performance, Heat Stress, Hepatic Transcriptome, Liver

## Abstract

**Objective:**

The objective of the present study was to investigate the effect of dietary betaine (BT) supplementation on the hepatic transcriptome profiles in broiler chickens raised under heat stress (HS) conditions.

**Methods:**

A total of 180 (21-d-old) Ross 308 male broiler chicks were allotted to 1 of 3 treatment groups with 6 replicated cages in a completely randomized design. One group was kept under thermoneutral conditions at all times and was fed a basal diet (PC). Other 2 groups were exposed to a cyclic heat stress condition. One of the 2 groups under heat stress conditions was fed the basal diet as a negative control (NC), whereas the other group was fed the basal diet supplemented with 0.2% BT. All chickens were provided with diets and water *ad libitum* for 21 d. Following the experiment, the liver samples were collected for RNA sequencing analysis.

**Results:**

Broiler chickens in NC and BT group had decreased (p<0.05) growth performance. In the transcriptome analysis, the number of differentially expressed genes were identified in the liver by HS conditions and dietary BT supplementation. In the comparison between NC and PC treatments, genes related to energy and nucleic acid metabolism, amino acid metabolism, and immune system were altered by HS, which support the reason why heat-stressed poultry had decreased growth performance. In the comparison between NC and BT treatments, genes related to lipid metabolism, carbohydrate metabolism, and immune system were differently expressed under HS conditions.

**Conclusion:**

HS negatively impacts various physiological processes, including DNA replication, metabolism of amino acids, lipids, and carbohydrates, and cell cycle progression in broiler chickens. Dietary BT supplementation, however, offers potential counteractive effects by modulating liver function, facilitating gluconeogenesis, and enhancing immune systems. These findings provide a basis for understanding molecular responses by HS and the possible benefits of dietary BT supplementation in broiler chickens exposed to HS.

## INTRODUCTION

Heat stress (HS) is a major concern in the poultry industry because it negatively affects productivity, health, and welfare of poultry. With increasing global temperatures, understanding the effects of HS on broiler chickens and finding effective ways to mitigate its negative outcomes are important to maintain the efficiency and sustainability of poultry production.

Broiler chickens are particularly susceptible to HS due to their rapid growth rate, high metabolic activity, and the relative inefficiency of heat dissipation mechanisms [1]. Therefore, broiler chickens exposed to HS experience various physiological disturbances including increased body temperature, respiration rate, and heart rate [1], which result in reduced feed intake (FI), decreased productive performance, impaired energy and lipid metabolism, and altered amino acid metabolism [2]. These negative outcomes of HS greatly increase the economic loss of poultry production. Based on a previous survey, HS resulted in annual economic losses of $128 to $165 million in US poultry industry at 2003 [3], suggesting that as the global warming progresses, this economic loss will be greatly elevated in the future.

Dietary supplementation of functional nutrients has been widely explored as a potential strategy to alleviate the negative outcomes of HS in broiler chickens. For instance, nutritional interventions using probiotics, vitamins, and electrolytes have shown to exert HS-reducing effects although the results are still inconsistent [4–6]. Among functional nutrients, betaine (BT) has gained attention due to its potential to improve growth performance, enhance nutrient utilization, and modulate stress responses in heat-stressed broiler chickens [7,8]. The BT can act as an osmolyte to preserve cell volume and fluid balance under HS conditions. Likewise, as a methyl donor, BT supports various molecular processes including protein synthesis and gene regulation, potentially alleviating the detrimental impacts of HS on the broiler chickens [9]. However, it is required to conduct more research regarding the molecular mechanisms how dietary BT supplementation exerts such a beneficial effect on broiler chickens exposed to HS.

Previous studies have primarily focused on apparent responses such as productive performance, stress responses, and welfare in broiler chickens exposed to HS with little consideration of the complex molecular mechanisms underlying these physiological responses. To overcome this limitation and gain a comprehensive understanding of the metabolic changes occurring in broiler chickens exposed to HS conditions, it is essential to investigate the physiological alterations at the molecular levels based on advanced techniques such as RNA sequencing (RNA-seq). Previous research utilizing RNA-Seq has shown that HS leads to elevated gene expression related to various nutrient metabolism and heat shock proteins in the liver of poultry [10,11]. The liver is often used as a target organ due to its high involvement in controlling nutrient metabolism, hormone synthesis, detoxification processes, immune system functions, and the regulation of circulatory protein levels in animals [12]. The HS induces metabolic imbalances in chickens, which the liver, being the center of nutrient metabolism and hormone regulation, actively tries to counterbalance [13]. Thus, understanding changes in liver functions under HS conditions is crucial in assessing the impact of HS on broiler chickens. In previous research, the effect of dietary BT supplementation has been investigated in stressed geese [14], specifically targeting the liver transcriptome. However, data regarding the effect of dietary BT supplementation on hepatic transcriptomic changes in broiler chickens exposed to HS have been limited.

Therefore, this study aimed to investigate the effect of dietary BT supplementation on the hepatic transcriptome profiles in broiler chickens raised under HS conditions.

## MATERIALS AND METHODS

### Animals, experimental design, and diets

The protocol for the current experiment was reviewed and approved by the Institutional Animal Care and Use Committee at Chung-Ang University (IACUC No. 2020-00022).

A total of 250 (1-d-old Ross) 308 male broiler chicks were obtained from a local hatchery (Dongsan broiler hatchery, Cheonan, Korea). All chicks were relocated to an environmentally controlled room and randomly assigned to each cage. Before the experiment commenced, birds were given a commercial diet containing adequate amounts of energy and nutrients. At 21 d of age, all chickens were weighed, and 70 chickens with extremely high and low body weight (BW) were excluded. The remaining 180 broiler chickens, with an average BW of 866±61.9 g, were assigned to one of three treatment groups, with six replicates per group. Each cage housed 10 broiler chickens. Chickens in one group were raised under thermoneutral (TN) conditions at 20°C and 57% relative humidity (RH) during a 21-d of overall experiment and were fed a basal diet (PC, positive control). The other two groups of chickens experienced a cyclic HS condition at 31°C to 32°C and 64% RH for 8 h daily, with the remaining time at 27°C to 28°C with 60% RH. Under this HS condition, birds in one group received the basal diet as a negative control (NC), whereas birds in the other group were given the basal diet supplemented with 0.2% BT (98%; Genebiotech, Gongju, Korea). The basal diet was formulated to meet or exceed the nutrient recommendations of the Ross 308 manual [15], and BT was added to the basal diet in the replacement of celite (Table 1). The supplemental level of BT in the present study was determined based on previous studies reporting its positive effects on poultry performance under HS conditions [16]. Broiler chickens were fed diets *ad libitum* for 21 d. A 23-h lighting program was used throughout the experiment. At the end of the experiment, final body weight (FBW), body weight gain (BWG), and FI were recorded. The feed conversion ratio (FCR) was calculated as FI divided by BWG after adjusting for mortality [16].

### Sample preparation for RNA-Seq

At the end of the experiment (i.e., 42 d of age), the individual weight of all chickens was recorded after an 8-h fasting. One bird with a BW close to the average BW in each replicated cage was selected for RNA-seq analysis in the liver. Therefore, a total of 18 birds (i.e., 6 birds per treatment) were selected and euthanized by CO_2_ asphyxiation. Liver samples were immediately collected, snap-frozen using liquid nitrogen, and stored at −80°C for total RNA isolation.

### RNA isolation, library construction, and RNA-Seq

Total RNA was extracted from the liver tissue using TRIzol reagent (Invitrogen, Life Technologies, Carlsbad, CA, USA), following the manufacturer’s instructions. Total RNA quality was assessed through the RNA-integrity number (RIN; Additional file 1: Supplementary Table S1). Total RNA was synthesized into cDNA for library construction. The constructed libraries were analyzed with an Illumina HiSeq X ten instrument (Illumina, Inc., San Diego, CA, USA), and paired-end (2×150 base pair) sequencing was performed. Detailed procedures for RNA sequencing were reported by Lim et al [17].

### Data processing and differentially expressed gene analyses

The initial step involved assessing the raw read data quality for each sample using the FastQC software v0.11.7. Any adaptors present in the reads were meticulously trimmed employing Trimmomatic v0.38, based on their quality results. These cleaned reads were then mapped to the GRCg6a reference genome (GCA_000002315.5) sourced from the Ensembl genome browser [18] using HISAT2 v2.1.0. We derived the raw counts for each library considering exons in Gallus gallus GTF v100 (Ensembl) with the assistance of the featureCounts tool from the Subread package v1.6.3.

For differential gene expression (DEG) analyses, we used edgeR v3.26.5. The raw counts underwent normalization through the trimmed mean of M-value technique. We pinpointed the DEGs in the livers of broiler chickens subjected to HS vs TN conditions by contrasting the NC vs PC treatment and further between the NC vs BT treatment under HS conditions. This identification relied on a set criteria: a false discovery rate of <0.05 coupled with an absolute log2 fold change of ≥1.

To visualize the gene expression disparities, we incorporated volcano plots and gene ontology (GO) tree maps. For the volcano plots, we juxtaposed the significance against the fold change of each gene, enabling a clear demarcation of highly regulated genes. Gene ontology tree maps were then generated to categorize the DEGs into specific biological processes and pathways, offering deeper insights into their functional roles.

Lastly, multidimensional scaling (MDS) plots were curated to showcase the relatedness between the samples for each tissue type. For a more comprehensive methodology, readers can refer to Lim et al [17].

### Functional enrichment analyses

The DEGs were annotated to GO terms and Kyoto encyclopedia of genes and genomes (KEGG) pathways based on the Database for Annotation, Visualization, and Integrated Discovery (DAVID) v6.8. The GO enrichment analyses were executed for biological process (BP), cellular component (CC), and molecular function (MF), applying cut-offs of p-value <0.1 and counts ≥2. Enriched GO terms were grouped with similar terms and visualized using treemaps with REVIGO. The most significant GO terms in each group were presented as representatives. The KEGG enrichment analyses were also conducted with the same cut-off criteria and represented by −log_10_ p-value and fold enrichment. All enrichment analyses were annotated for *Gallus gallus* species.

### Quantitative real-time polymerase chain reaction validation

To verify the reliability of the expression profiles of RNA-seq data, we randomly evaluated the expression of twelve each for DEGs by quantitative real-time polymerase chain reaction (qPCR) followed the method of Kim et al [13]. In short, total RNA was extracted from the liver using TRIzol reagent (Invitrogen, USA) according to the manufacturer’s instructions. Gene expression was examined for minichromosome maintenance complex component 3 (*MCM3*), arachidonate 5-lipoxygenase (*ALOX5*), xanthine dehydrogenase (*XDH*), prostaglandin D2 synthase 21 kDa (brain) (*PTGDS*), interleukin 4 induced 1 (*IL4I1*), perilipin 1 (*PLIN1*), acetyl-CoA carboxylase alpha (*ACACA*), and glucosaminyl (N-acetyl) transferase 2 (*GCNT2*) in the hepatic tissues. Gene-specific primers for target genes were designed using NCBI/Primer-BLAST. The primer sequences and amplification temperatures are listed in Supplementary Table S2. The relative quantification of gene-specific expression was calculated using the 2^−ΔΔCt^ method after normalization to glyceraldehyde-3-phosphate dehydrogenase (*GAPDH*) [13].

### Statistical analysis

Data for growth performance were analyzed using analysis of variance as a completely randomized design with the PROC MIXED procedure (SAS Institute Inc., Cary, NC, USA). The replicate served as an experimental unit for all measurements. Outlier data were examined using the PROC UNIVARIATE procedure of SAS; however, no outliers were detected. The LSMEANS procedure was employed to compute treatment means, and the PDIFF option of SAS was used for the mean separation if the difference was significant. Statistical significance was considered at p<0.05.

## RESULTS

### Growth performance

The growth performance including FBW, BWG, and FI was reduced (p<0.05) in NC treatment compared to PC treatment (Table 2). Birds in BT treatment showed increased BWG and FI compared to NC treatment, although significance was not identified. Neither environmental conditions nor dietary BT supplementation under HS conditions had a significant impact on the FCR of broiler chickens.

### Data processing and transcriptomes analysis

A total of 401 million paired-end reads were generated from 18 liver samples. The average number of generated reads per sample was 22.3 million and the average trimmed read was 21.8 million (2.06% were trimmed out; Supplementary Table S3). Overall mapping rates ranged from 94.38% to 97.00% and the average unique mapping rate was 89.51%.

**Figure 1:**
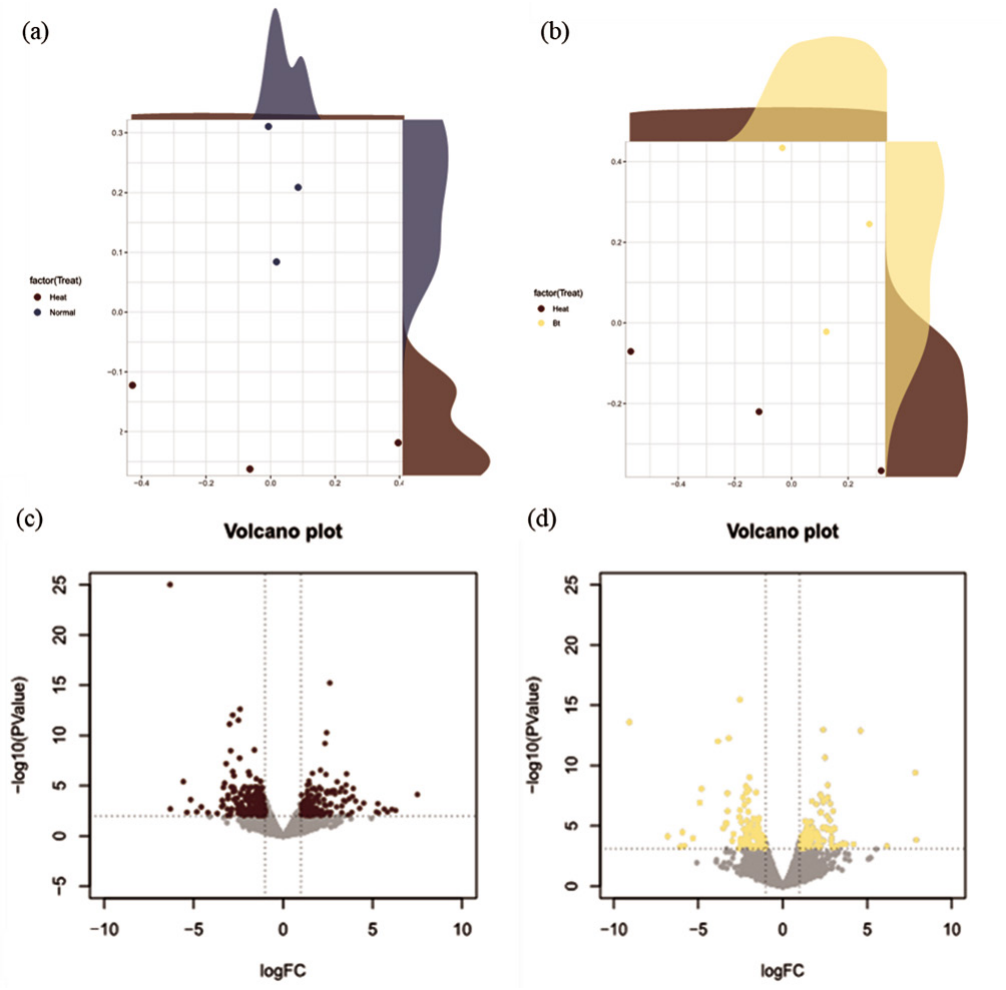
Multidimensional scaling (MDS) reveals separate clusters in the two comparisons based on the transcriptomes under different conditions. (a) The MDS plot displays the distinction between positive control (PC) and negative control (NC), whereas (b) compares negative control (NC) and betaine (BT) treatment; PC, thermoneutral conditions, NC and BT, heat stress conditions. (c) Shows a volcano plot indicating significant differently expressed genes (DEGs) in the positive control (brown) compared to NC, whereas (d) displays a volcano plot but for betaine treatment (yellow) compared to NC. The x and y axes of the volcano plots show the log_2_ fold changes and −log_10_ p-values, respectively.

The NGS reads were generated from the liver tissue for HS conditions (i.e., NC and BT treatment; n = 6) or TN conditions (i.e., PC treatment; n = 6). The NGS reads, derived from liver tissues exposed to HS conditions, provide a detailed snapshot of the transcriptional landscape under stress. In the MDS analysis, three samples with high density were selected and displayed to show clear clustering for the liver tissue (Figure 1a, 1b). The analysis of DEGs was performed by comparing gene expression levels in either PC treatment or BT treatment with those in NC treatment and was visualized as a volcano plot (Figure 1c, 1d). In comparison with NC treatment, PC treatment showed 79 up-regulated DEGs and 58 down-regulated DEGs, whereas BT treatment displayed 107 up-regulated DEGs and 99 down-regulated DEGs.

### Functional annotations

**Figure 2:**
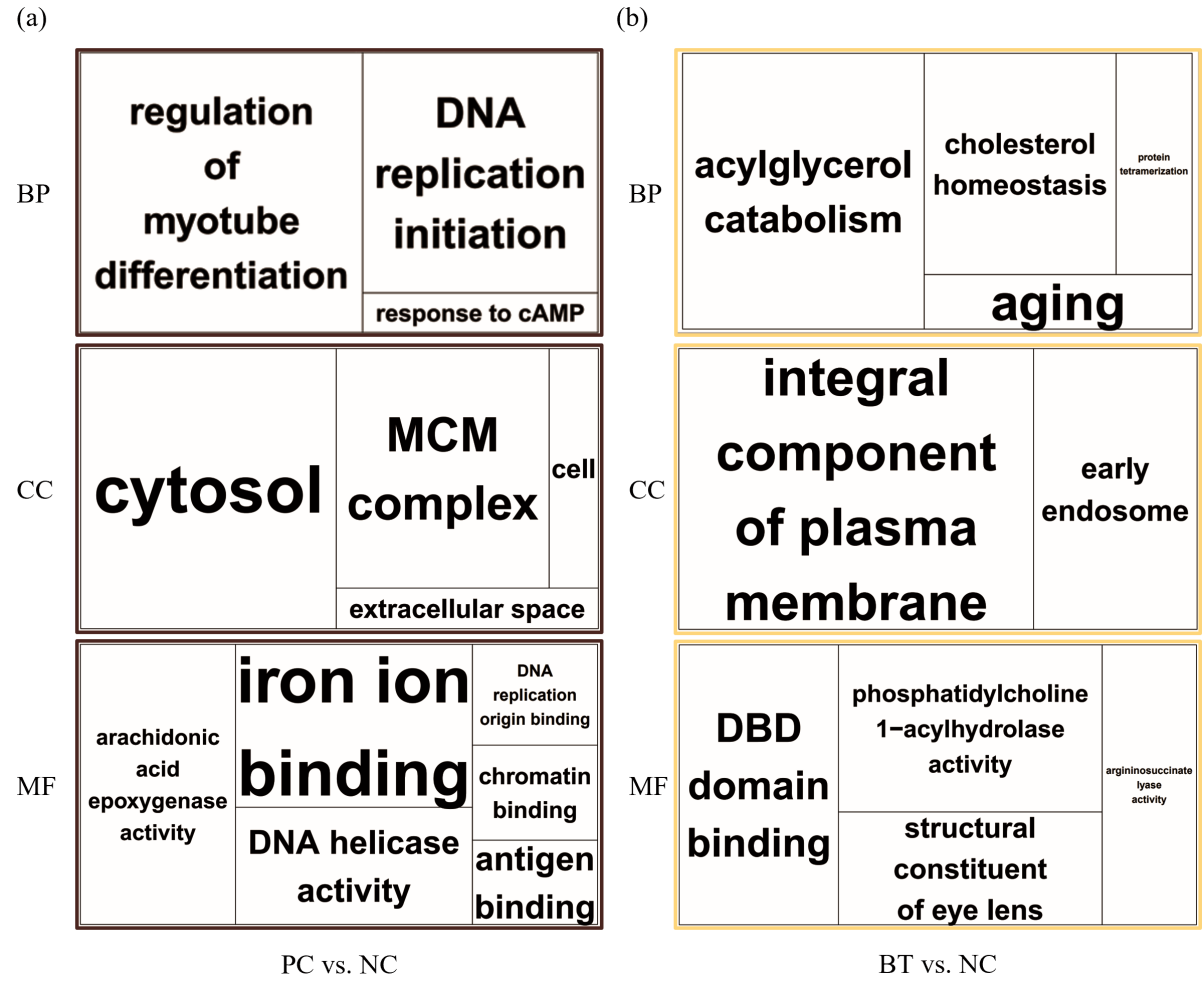
Functional enrichment in the liver is depicted in the gene ontology treemaps, built upon the p-values associated with biological process (BP), cellular component (CC), and molecular function (MF) terms. (a) Corresponds to the positive control (brown), whereas figure (b) pertains to the betaine treatment (yellow).

**Figure 3:**
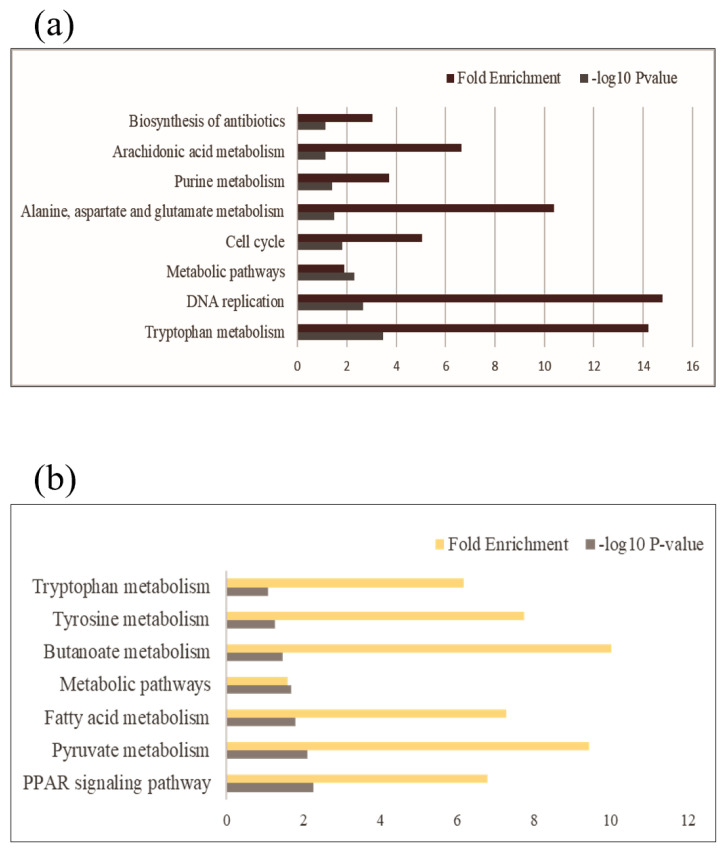
The Kyoto encyclopedia of genes and genomes (KEGG)-enriched pathways for functional enrichment in the liver are displayed in (a) and (b). (a) Shows the enriched pathways in the positive control (brown) with -log_10_ p-values >1.0, whereas (b) displays the same for the betaine treatment (yellow).

Enrichment analyses were performed based on the GO (Figure 2) and KEGG databases in the liver. The BPs of GO for comparison between NC and PC treatment were significantly enriched to ‘*regulation of myotube differentiation*’, ‘*DNA replication initiation*’, and ‘*response to cAMP*’. The CCs of GO for comparison between NC and PC treatment were enriched to ‘*cytosol*’, ‘*MCM complex*’, ‘*cell*’, and ‘*extracellular space*’. The MFs of GO for comparison between NC and PC treatment were enriched to ‘*arachidonic acid epoxygenase activity’*, ‘*iron ion binding*’, ‘*DNA helicase activity*’, ‘*DNA replication origin binding*’, ‘*chromatin binding*’, and ‘*antigen binding*’. In the comparison between NC and BT treatments, the BPs of GO were enriched to ‘*acylglycerol catabolism*’, ‘*cholesterol homeostasis*’, ‘*protein tetramerization’*, and ‘*aging*’. The CCs of GO were enriched to ‘*integral component of plasma membrane*’ and ‘*early endosome*’. The MFs of GO were enriched to ‘*DBD domain binding’*, ‘*phosphatidylcholine 1–acylhydrolase activity*’, ‘*structural constituent of eye lens*’, and ‘*argininosuccinate lyase activity*’. In a similar pattern to GO terms, the KEGG pathways for the comparison between NC and PC treatment were identified with ‘*Biosynthesis of antibiotics’*, ‘*Arachidonic acid metabolism*’, ‘*Purine metabolism*’, ‘*Alanine, aspartate and glutamate metabolism’*, ‘*Cell cycle*’, ‘*Metabolic pathways*’, ‘*DNA replication*’, and ‘*Tryptophan metabolism*’ (Figure 3a). The KEGG pathways for the comparison between NC and BT treatment were identified with ‘*Tryptophan metabolism*’, ‘*Tyrosine metabolism*’, ‘*Butanoate metabolism’*, ‘*Metabolic pathways*’, ‘*Fatty acid metabolism*’, ‘*Pyruvate metabolism*’, and ‘*PPAR signaling pathway*’ (Figure 3b).

### Quantitative real-time polymerase chain reaction validation of gene expression

**Figure 4:**
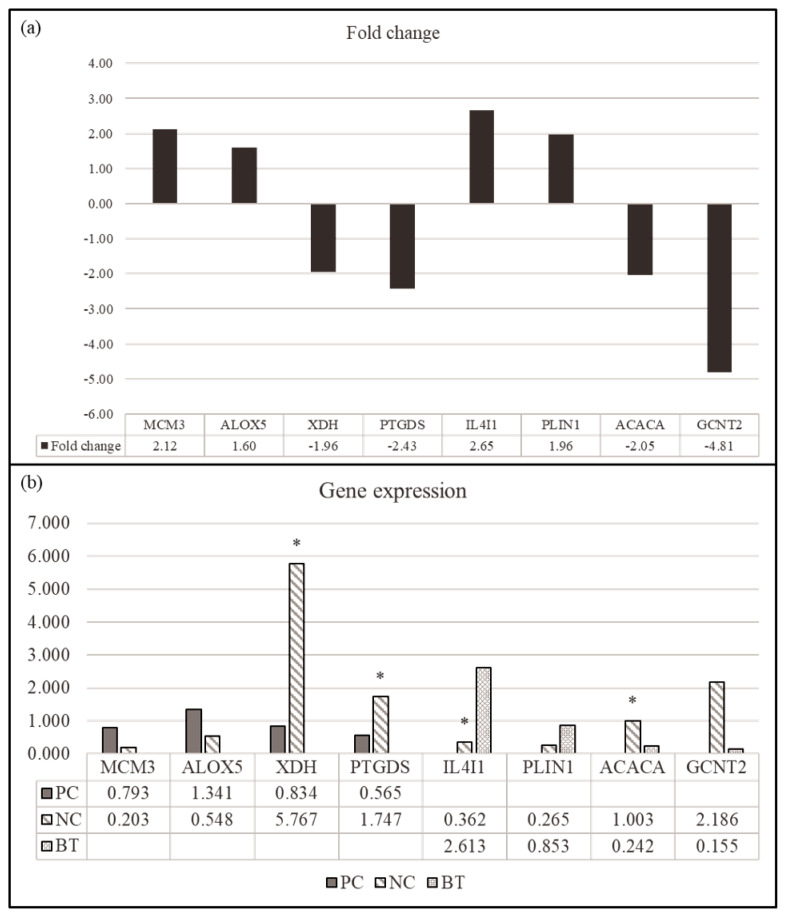
Validation of the differentially expressed genes (DEGs) by RT-PCR (n = 6). (a) Comparison (log_2_ fold change) of the RNA-seq data of PC (MCM3, ALOX5, XDH, and PTGDS) and BT treatment (IL4I1, PLIN1, ACACA, and GCNT2) relative to NC treatment. (b) Individual variability of validated DEGs in RT-PCR in the comparison between NC and PC treatments and between NC and BT treatments. PC, positive control; NC, negative control; BT, basal diet + 0.2% betaine. * indicates p<0.05.

We selected four DEGs (*MCM3*, *ALOX5*, *XDH*, and *PTGDS*) for comparison between NC and PC treatment in the hepatic tissues and four DEGs (*IL4I1*, *PLIN1*, *ACACA*, and *GCNT2*) in the comparison between NC and BT treatments in the hepatic tissues for the validation by qPCR (Figure 4). The genes were selected to identify those within the relevant KEGG pathway that demonstrated the most significant fold change. All genes showed a similar expression pattern in both of qPCR and RNA-seq. These results demonstrated the reliability of our RNA-seq data and confirmed the accuracy of the identified transcripts.

## DISCUSSION

Broiler chickens raised under HS conditions have been reported to experience a range of abnormal nutrient metabolism in the body [13,19], which leads to negative outcomes in productive performance, health, and welfare of poultry [20, 21]. Similar results regarding decreased growth performance of broiler chickens were observed in the current study. The impaired performance in poultry exposed to HS has been largely attributed to the loss of appetite [22], decreased digestion, absorption, and utilization of dietary nutrients and energy [23,24], endocrine disorders, systemic immune dysregulation, abnormal behavior, and increased oxidative stress [25–27]. Numerous efforts have been made to minimize this negative impact on poultry performance through dietary supplementation of various functional nutrients. BT has been widely used as a dietary supplement to ameliorate the negative effect on poultry performance because of its relatively consistent effects [7]. The beneficial effect of BT was also observed in this study although we failed to find the significance.

The liver is a multifunctional organ involved in the bile secretion, nutrient metabolism, and detoxification [12]. Additionally, the liver plays a role in immune responses by producing acute-phase proteins during high-inflammatory insults [28]. Consequently, the liver is considered a primary target metabolic organ as affected by various intrinsic animal factors and external factors such as the environment and dietary treatment. In the current study, we carefully selected the representative liver samples from each treatment based on the high density of three individuals in the MDS plot analysis, ensuring the reliability and accuracy of the transcriptome analysis. The distinct separation between treatments in the transcriptomic data highlights the effectiveness of the current experimental design and supports the credibility of the findings. As a result, our research presents a reliable and robust foundation for understanding the hepatic molecular responses of broiler chickens to environmental treatments and dietary BT supplementation.

### Comparison between NC and PC treatment: energy and nucleic acid metabolism-related pathways

Based on the liver transcriptome analysis, broiler chickens exposed to HS conditions demonstrated alterations in energy and nucleic acid metabolism. This functional change could account for our observation of decreased growth performance as well as previous results on altered energy metabolism [29], and the reduced ability to recover cell cycle and DNA replication in broiler chickens under HS conditions [30].

**Figure 5:**
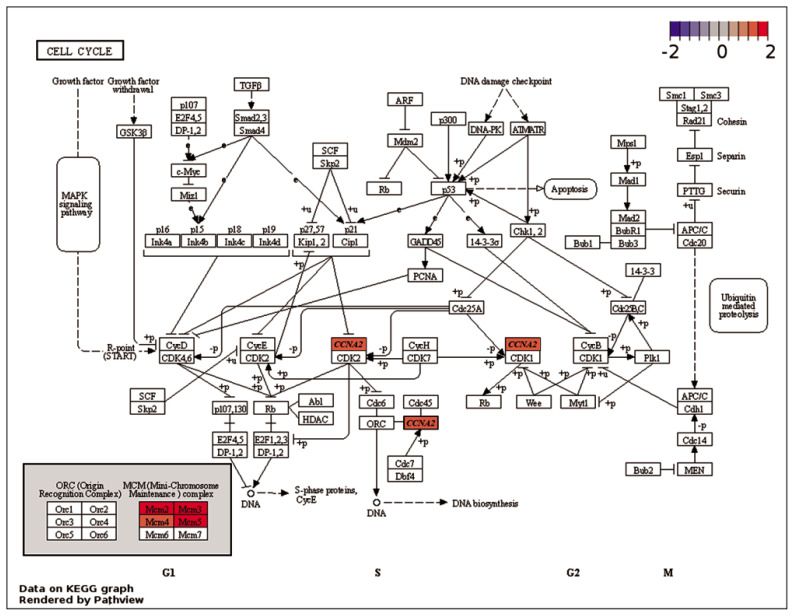
Gene modulations of the liver tissue in the ‘cell cycle’ pathway are presented as log_2_ fold change values for the comparison between positive control (PC) and negative control (NC).

**Figure 6:**
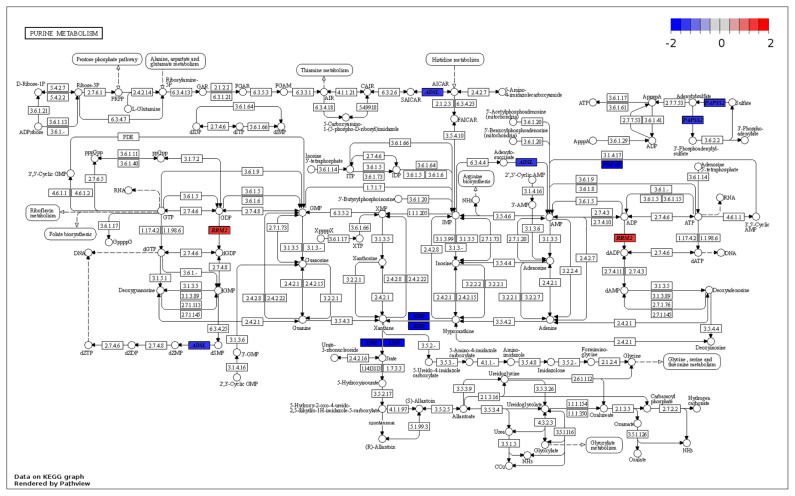
Gene modulations of the liver tissue in the ‘purine metabolism’ pathway are presented as log_2_ fold change values for the comparison between positive control (PC) and negative control (NC).

The expression levels of various genes connected to ‘*DNA replication*’ (Supplementary Figure S1) and ‘*Cell cycle*’ (Figure 5) were altered due to HS conditions. As compared to NC treatment, the minichromosome maintenance complex component (MCM) proteins, including *MCM2*, *MCM3*, *MCM4*, and *MCM5* genes, exhibited up-regulation in PC treatment. These MCM proteins play a crucial role in initiating eukaryotic genome replication and have distinct functions in DNA replication [31,32]. Therefore, the up-regulation of these genes may indicate that HS causes damage or reduction in the DNA replication process. Cyclin A2 (*CCNA2*) is crucial for cell cycle regulation, primarily functioning during the S phase and G2/M transition [33]. When activated by binding to CDK, it promotes cell cycle progression and DNA replication, contributing to normal cell division, growth, and tissue development in organisms. Additionally, *CCNA2* contributes to the prevention of abnormal changes that could impact cellular development and function by regulating the cell cycle [33]. In the present study, the *CCNA2* gene was up-regulated in PC treatment as compared to NC treatment, suggesting that HS may have a detrimental effect on cell division and growth in the liver. Moreover, the expression levels of genes involved in the ‘*Purine metabolism*’ pathway (Figure 6), including adenylosuccinate lyase (*ADSL*), xanthine dehydrogenase (*XDH*), 3′-phosphoadenosine 5′-phosphosulfate synthase 2 (*PAPSS2*), and phosphodiesterase 4B (*PDE4B*), were down-regulated in PC treatment. The *ADSL* is involved in purine metabolism and catalyzes two non-sequential steps in the de novo purine nucleotide biosynthesis. It converts adenylosuccinate to adenosine monophosphate (AMP) and fumarate, as well as inosine monophosphate (IMP) to xanthosine monophosphate (XMP) [34]. The *XDH* enzyme maintains the balance of purine nucleotide pools in cells [35]. The *PAPSS2* synthesizes PAPS, essential for sulfotransferase reactions and sulfation of various molecules [36]. The *PDE4B* regulates intracellular levels of cAMP and modulates cell functions [37]. Compared to HS conditions, the down-regulation of *ADSL*, *XDH*, *PAPSS2*, and *PDE4B* genes in TN conditions may indicate that there was less damage to energy generation, signal transmission, and nucleic acid component production, making these pathways less crucial and leading to their down-regulation [38]. The down-regulation of purine metabolism-related genes in PC treatment may imply that energy production and cellular signaling are less affected, allowing for sufficient energy supply and proper information exchange within cells and the organism [39].

### Comparison between NC and PC treatment: amino acid metabolism-related pathways

Genes involved in ‘*tryptophan metabolism*’ (Supplementary Figure S2), exhibited altered expression in response to HS. 3-Hydroxyanthranilate 3,4-Dioxygenase (*HAAO*) is a gene involved in tryptophan metabolism. *HAAO* plays a role in the breakdown of tryptophan, contributing to the production of niacin (vitamin B3). The down-regulation of HAAO expression under TN conditions, as observed in this study, may suggest that tryptophan metabolism and niacin production are less affected compared to the HS conditions. In NC treatment, the gene expression is likely increased to alleviate the impacts on energy metabolism, cellular repair, and antioxidative processes, leading to a relative down-regulation under TN conditions. Aminocarboxymuconate semialdehyde decarboxylase (*ACMSD*) is another gene involved in tryptophan metabolism. This protein degrades aminocarboxymuconate semialdehyde, an intermediate product in tryptophan metabolism, and regulates the kynurenine and quinolinic acid pathways, which are known to influence neural activity in the central nervous system [40]. The down-regulation of ACMSD expression in TN conditions, as observed in this study, may suggest that under normal temperature conditions, crucial pathways in tryptophan metabolism are not influenced, potentially maintaining neural cell function and survival [40]. In contrast, chickens exposed to HS conditions had an increased requirement for energy and amino acids [13], leading to an up-regulation of ACMSD expression to meet this requirement. The down-regulation of these genes compared to the HS group implies that chickens under TN conditions may not require as much involvement of amino acid metabolism to cope with environmental stressors, allowing for adequate energy metabolism, cellular repair, antioxidative processes, and neural function. Specifically, balanced amino acid metabolism may support protein synthesis and tissue formation that are essential for chicken growth and development, positively affecting overall health and productivity.

### Comparison between NC and PC treatment: immune system-relative pathways

Transcriptomic analysis revealed that the ‘*Arachidonic acid metabolism*’ pathway (Supplementary Figure S3) was identified when comparing broiler chickens raised under HS conditions (NC) to those raised under TN conditions (PC). In poultry, the arachidonic acid pathway refers to the series of enzymatic reactions that convert arachidonic acid into eicosanoids, including prostaglandins, thromboxanes, and leukotrienes [41]. These eicosanoids have a wide range of functions in chickens, such as inflammation and immune response, cellular signaling, reproduction, cardiovascular and renal functions, and gastrointestinal function [41].

In ‘*Arachidonic acid metabolism*’ pathway (Supplementary Figure S3), three genes were altered by HS conditions. Arachidonate 5-lipoxygenase (*ALOX5*), leukotriene C4 synthase (*LTC4S*), and prostaglandin D2 synthase (*PTGDS*) are genes associated with the metabolism of arachidonic acid and the production of eicosanoids, which are lipid mediators involved in various physiological processes, including inflammation, immune response, and cellular signaling [42]. The up-regulation of *ALOX5* in PC treatment indicates an increase in the production of leukotrienes and other eicosanoids. Leukotrienes play a role in inflammatory and immune responses, which can be activated under stress conditions to protect cells and tissues from damage. The up-regulation of ALOX5 in TN conditions could be interpreted as a relative increase due to the weakening of the defense mechanism against the detrimental effects of HS in NC treatment group.

On the other hand, *LTC4S* and *PTGDS*, which are involved in the synthesis of leukotriene C4 and prostaglandin D2, respectively, were down-regulated in PC treatment. It has been reported that HS conditions can affect immune responses in the liver, with increased inflammatory responses in hepatic tissues potentially impairing the hepatic immune responses of heat-stressed animals [13]. The down-regulation of these genes in PC treatment may suggest that broiler chickens exposed to HS experience an increase in inflammatory responses, leading to the up-regulation of genes to mitigate these effects. This could imply a potential imbalance in the production of eicosanoids, which may negatively affect overall inflammatory and immune responses in chickens. This imbalance could result in a reduced ability to cope with stressors, leading to decreased health and productivity. These changes in the expression of *ALOX5*, *LTC4S*, and *PTGDS* in PC treatment may serve as counter-evidence, suggesting that HS could potentially impact eicosanoid metabolism associated with inflammatory and immune responses in chickens.

### Comparison between NC and betaine treatment: lipid metabolism pathway

**Figure 7:**
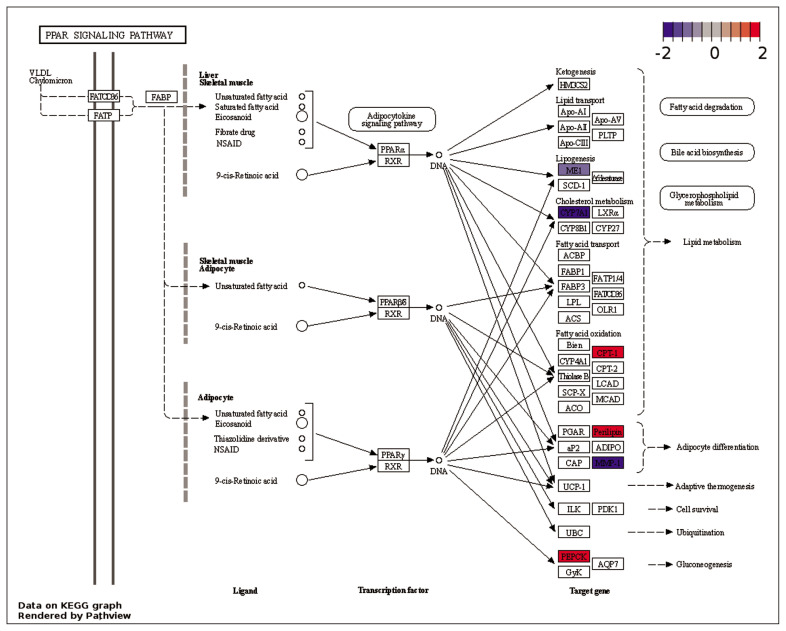
Gene modulations of the liver tissue in the ‘PPAR signaling pathway’ are presented as log_2_ fold change values for the comparison between negative control (NC) and betaine treatment (BT).

In comparison to NC treatment, the BT treatment displayed the activation of ‘*PPAR signaling pathway*’ (Figure 7). The peroxisome proliferator-activated receptors (PPARs) are a group of nuclear receptor proteins that function as transcription factors regulating the expression of genes. They play a role in the cellular regulation of fatty acid metabolism [43]. In ‘*PPAR signaling pathway*’ (Figure 7), carnitine palmitoyl transferase 1A (*CPT1A*) was up-regulated in BT treatment, which indicates an increase in fatty acid oxidation. *CPT1A* is responsible for transporting long-chain fatty acids into the mitochondrial matrix for β-oxidation [44]. The up-regulation of perilipin 1 (*PLIN1*) also indicates improved lipid metabolism, as *PLIN1* plays a role in protecting lipid droplets from lipolysis. The up-regulation of acetyl-CoA acetyltransferase 2 (*ACAT2*) and the down-regulation of acetyl-CoA carboxylase alpha (*ACACA*) genes suggest an impact on acetyl-CoA metabolism, which plays a crucial role in lipid and energy metabolism [45]. Both *ACAT2* and *ACACA* are involved in the metabolism of acetyl-CoA, a key intermediate in various metabolic pathways [46]. *ACAT2* acts as an acetyl-CoA acetyltransferase, converting acetyl-CoA to acetoacetyl-CoA, which plays a crucial role in ketone body formation, cholesterol synthesis, and fatty acid elongation [46]. *ACACA* functions as an acetyl-CoA carboxylase, converting acetyl-CoA to malonyl-CoA, a key intermediate in fatty acid synthesis [47]. Integrating these results, it can be inferred that dietary BT supplementation in broiler chickens exposed to HS may increase ketone body production and cholesterol synthesis but decrease fatty acid synthesis. In previous studies, BT has been utilized as a lipotropic substance to facilitate the breakdown and release of fat from the liver, and promote lipid oxidation in the body [48].

### Comparison between NC and betaine treatment: carbohydrate metabolism pathways

In ‘*Pyruvate metabolism*’ (Supplementary Figure S4), phosphoenolpyruvate carboxykinase 1 (*PCK1*) was up-regulated in BT treatment. *PCK1* is an enzyme involved in the gluconeogenesis process, converting non-carbohydrate precursors, including amino acids, lactate, and glycerol into glucose [8]. This process plays a vital role in regulating blood glucose levels depending on energy requirements. The up-regulation of *PCK1* suggests an increase in gluconeogenesis activity by dietary BT supplementation, which may aid in maintaining blood glucose levels in broiler chickens exposed to HS conditions.

In ‘*Metabolic pathway’*, the up-regulation of beta-1,3-N-acetylglucosaminyltransferase 2 (*B3GNT2*) and down-regulation of glucosaminy (N-acetyl) transferase 2, I-branching enzyme (*GCNT2*) genes in BT treatment suggest that BT may have a regulatory effect on these genes, which could potentially impact growth performance. The *B3GNT2* gene is involved in the synthesis of complex carbohydrates, including glycoproteins and glycolipids, which play essential roles in various biological processes such as cell adhesion, cell signaling, and immune response [49]. The up-regulation of *B3GNT2* may indicate that dietary BT supplementation enhances these processes in heat-stressed chickens, potentially leading to improved growth performance. Despite few available data specifically for broiler chickens exposed to HS conditions, the observed changes in the *B3GNT2* gene expression may potentially support the HS-reducing effects of dietary BT supplementation in previous studies [7–9,26]. The *GCNT2* gene is responsible for the synthesis of complex carbohydrates in the golgi apparatus, specifically for the formation of I-branches in certain types of glycans [50]. Down-regulation of the *GCNT2* may suggest that dietary BT supplementation reduces the formation of I-branches in heat-stressed chickens, possibly altering carbohydrate structure and function. This alteration may also influence growth performance in broiler chickens raised under HS conditions. It is important to note that these results should be further investigated to understand the exact mechanisms and implications of dietary BT supplementation on the regulation of *B3GNT2* and *GCNT2* genes in broiler chickens exposed to HS conditions, particularly with regard to their productive performance.

### Comparison between NC and betaine treatment: immune system-related pathway

In ‘*Tyrosine metabolism*’ (Supplementary Figure S5) and ‘*Tryptophan metabolism*’ (Supplementary Figure S6) pathway, interleukin 4 induced 1 (*IL4I1*) was up-regulated in BT treatment. *IL4I1* is an enzyme involved in the catabolism of aromatic amino acids, specifically L-phenylalanine [51,52]. The up-regulation of *IL4I1* may suggest modulation of immune responses and potential anti-inflammatory effects in heat-stressed chickens through dietary BT supplementation. In a study by Lee et al [51], broiler chickens exposed to acute HS showed up-regulation of *IL4I1*, indicating a modulation of immune responses and potential anti-inflammatory effects. This observation suggests that dietary BT supplementation could play a beneficial role in mitigating the adverse effects of HS on the immune systems of broiler chickens.

Although it was not differentiated by KEGG pathway analysis, different expression levels of immune system-related genes were also found in BT treatment. Under HS conditions, dietary BT supplementation resulted in the up-regulation of C-X-C motif chemokine receptor 4 (*CXCR4*), defensin beta 4A (*DEFB4A*), and cysteine-rich secretory protein 3 (*CRISP3*) genes, whereas the interleukin 7 receptor (*IL7R*) gene was down-regulated. The up-regulation of *CXCR4* may suggest an increased response to chemokines, which are involved in immune cell trafficking and inflammation [53]. *DEFB4A* is an antimicrobial peptide [54], and its up-regulation may imply enhanced innate immune defense mechanisms. *CRISP3* is a protein involved in immune response and inflammation [55], and its up-regulation could indicate an increased capacity for managing inflammatory processes. On the other hand, the down-regulation of the *IL7R* could suggest a modulation of the adaptive immune response, as *IL7R* is involved in lymphocyte development and homeostasis [56]. Overall, these gene expression changes may indicate a complex interplay between innate and adaptive immune responses in heat-stressed chickens fed diets supplemented with BT. However, a comprehensive understanding of the impact of these gene regulations requires further investigation.

## CONCLUSION

This study provides valuable insights into transcriptional changes in the liver of broiler chickens and the impact of BT supplementation on transcriptional changes under HS conditions. HS disrupts the physiological processes including DNA replication, various metabolic functions, and requirements of energy and amino acid. More notably, our findings reveal that dietary BT supplementation serves as a potent countermeasure against these detrimental effects of HS by regulating liver function such as improved lipid metabolism, glucose management, and immune response. The current findings offer a foundation for understanding the underlying mechanisms of HS response and the potential benefits of dietary BT supplementation in broiler chickens under HS conditions. Future research is required to verify the physiological and metabolical alterations in broiler chickens by dietary BT supplementation under HS conditions through integrated transcriptomic analysis among various pivotal tissues in the body.

## Figures and Tables

**Table 1:** Composition and nutrient concentration of the basal diet

Items	Basal diet
Ingredients (%)	
Corn	58.71
Soybean meal (46% crude protein)	24.47
Corn gluten meal	5.77
Tallow	5.56
Mono-dicalcium phosphate	1.48
Limestone	1.21
DL-Methionine (98%)	0.32
L-Lysine HCl (99%)	0.58
L-Threonine (98.5%)	0.10
Choline (50%)	0.10
NaHCO_3_	0.10
Salt	0.20
Vitamin premix^1)^	0.10
Mineral premix^2)^	0.10
Celite	1.20
Total	100.00
Calculated energy and nutrient	
AME_n_ (kcal/kg)	3,200
Crude protein (%)	19.54
Digestible lysine (%)	1.04
Digestible arginine (%)	1.10
Digestible tryptophan (%)	0.18
Digestible methionine + cysteine (%)	0.80
Digestible methionine (%)	0.54
Digestible threonine (%)	0.69
Total calcium (%)	0.79
Available phosphorus (%)	0.40

AME_n_, nitrogen-corrected apparent metabolizable energy.

1) Provided per kg of the complete diet: vitamin A, 12,000 IU (retinyl acetate); vitamin D_3_, 4,000 IU; vitamin E, 80 mg; vitamin K_3_, 4.0 mg (menadione dimethpyrimidinol); vitamin B_1_, 4.0 mg; vitamin B_2_, 10 mg; vitamin B_6_, 6.0 mg; vitamin B_12_, 20.0 μg; calpan 20 mg; folic acid, 2.0 mg; biotin, 200 μg; niacin, 60 mg; antioxidant 2.0 mg; toyouserin 10.0 mg.

2) Provided per kg of the complete diet: manganese, 120 mg (MnO); copper 16.0 mg (CuSO_4_); zinc 100 mg (ZnSO_4_); iron, 60 mg (FeSO_4_); iodine, 1.25 mg (Ca[IO_3_]_2_]; cobalt, 1.0 mg; selenium, 300 μg.

**Table 2:** Effect of dietary betaine supplementation on growth performance of broiler chickens raised under heat stress conditions

Environment	Diet^1)^	Growth performance			

		FBW (kg)	BWG (kg)	FI (kg)	FCR (kg/kg)
TN	Basal (PC)	2.75^a^	1.89^a^	3.23^a^	1.71
HS	Basal (NC)	2.39^b^	1.52^b^	2.62^b^	1.72
	BT	2.39^b^	1.53^b^	2.63^b^	1.72
SEM		0.275	0.269	0.495	0.026
p-value		<0.01	<0.01	<0.01	0.95

Data are least squares means of 6 observations per treatment.

FBW, body weight; BWG, body weight gain; FI, feed intake; FCR, feed conversion ratio; TN, thermoneutral condition; HS, heat stress condition; SEM, standard error of the means.

1) PC, basal diet; NC, basal diet; BT, basal diet+0.2% betaine.

a,b Means in the same column with different superscripts are different (p<0.05).

## SUPPLEMENTARY MATERIAL

**Supplementary Table S1**. RNA quality score of broiler chickens raised under thermoneutral (TN) or heat stress (HS) conditions

**Supplementary Table S2**. Primers used for quantitative RT-PCR

**Supplementary Table S3**. Overview of data processing of broiler chickens in current experiment

**Supplementary Figure S1**. Gene modulations of the liver tissue in the ‘DNA replication’ pathway are presented as log_2_ fold change values for the comparison between positive control (PC) and negative control (NC).

**Supplementary Figure S2**. Gene modulations of the liver tissue in the ‘tryptophan metabolism’ pathway are presented as log_2_ fold change values for the comparison between positive control (PC) and negative control (NC).

**Supplementary Figure S3**. Gene modulations of the liver tissue in the ‘arachidonic acid metabolism’ pathway are presented as log_2_ fold change values for the comparison between positive control (PC) and negative control (NC).

**Supplementary Figure S4**. Gene modulations of the liver tissue in the ‘pyruvate metabolism’ are presented as log_2_ fold change values for the comparison between negative control (NC) and betaine treatment (BT).

**Supplementary Figure S5**. Gene modulations of the liver tissue in the ‘tyrosine metabolism’ are presented as log_2_ fold change values for the comparison between negative control (NC) and betaine treatment (BT).

**Supplementary Figure S6**. Gene modulations of the liver tissue in the ‘tryptophan metabolism’ are presented as log_2_ fold change values for the comparison between negative control (NC) and betaine treatment (BT).

## References

[1] He SP, Arowolo MA, Medrano RF (2018). Impact of heat stress and nutritional interventions on poultry production. Worlds Poult Sci J.

[2] Balnave D (2004). Challenges of accurately defining the nutrient requirements of heat-stressed poultry. Poult Sci.

[3] St-Pierre NR, Cobanov B, Schnitkey G (2003). Economic losses from heat stress by US livestock industries. J Dairy Sci.

[4] Lin H, Du R, Gu XH, Li FC, Zhang ZY (2000). A study on the plasma biochemical indices of heat-stressed broilers. Asian-Australas J Anim Sci.

[5] Lan PTN, Sakamoto M, Benno Y (2004). Effects of two probiotic *Lactobacillus* strains on jejunal and cecal microbiota of broiler chicken under acute heat stress condition as revealed by molecular analysis of 16S rRNA genes. Microbiol Immunol.

[6] Kucuk O, Sahin N, Sahin K (2003). Supplemental zinc and vitamin A can alleviate negative effects of heat stress in broiler chickens. Biol Trace Elem Res.

[7] Saeed M, Babazadeh D, Naveed M, Arain MA, Hassan FU, Chao S (2017). Reconsidering betaine as a natural anti-heat stress agent in poultry industry: A review. Trop Anim Health Prod.

[8] Al-Sagan AA, Al-Abdullatif A, Hussein EOS (2021). Effects of betaine supplementation on live performance, selected blood parameters, and expression of water channel and stress-related mRNA transcripts of delayed placement broiler chicks. Front Vet Sci.

[9] Ratriyanto A, Mosenthin R (2018). Osmoregulatory function of betaine in alleviating heat stress in poultry. J Anim Physiol Anim Nutr.

[10] Xie J, Tang L, Lu L (2014). Differential expression of heat shock transcription factors and heat shock proteins after acute and chronic heat stress in laying chickens (*Gallus gallus*). PloS One.

[11] Jastrebski SF, Lamont SJ, Schmidt CJ (2017). Chicken hepatic response to chronic heat stress using integrated transcriptome and metabolome analysis. PloS One.

[12] Zaefarian F, Abdollahi MR, Cowieson A, Ravindran V (2019). Avian liver: the forgotten organ. Animals.

[13] Kim DY, Lim B, Kim J, Kil DY (2022). Integrated transcriptome analysis for the hepatic and jejunal mucosa tissues of broiler chickens raised under heat stress conditions. J Anim Sci Biotechnol.

[14] Yang Z, Yang HM, Gong DQ (2018). Transcriptome analysis of hepatic gene expression and DNA methylation in methionine- and betaine-supplemented geese (Anser cygnoides domesticus). Poult Sci.

[15] Aviagen (2018). Ross 308 broiler: nutrition specifications.

[16] Kim DY, Kim JH, Choi WJ, Han GP, Kil DY (2021). Comparative effects of dietary functional nutrients on growth performance, meat quality, immune responses, and stress biomarkers in broiler chickens raised under heat stress conditions. Anim Biosci.

[17] Lim B, Kim S, Lim K (2020). Integrated time-serial transcriptome networks reveal common innate and tissue-specific adaptive immune responses to PRRSV infection. Vet Res.

[18] Ensembl (c2023). Gallus_gallus – Ensembl genome browser 105 [internet].

[19] Wasti S, Sah N, Mishra B (2020). Impact of heat stress on poultry health and performances, and potential mitigation strategies. Animals.

[20] Habashy WS, Milfort MC, Adomako K, Attia YA, Rekaya R, Aggrey SE (2017). Effect of heat stress on amino acid digestibility and transporters in meat-type chickens. Poult Sci.

[21] Habashy WS, Milfort MC, Fuller AL, Attia YA, Rekaya R, Aggrey SE (2017). Effect of heat stress on protein utilization and nutrient transporters in meat-type chickens. Int J Biometeorol.

[22] Bortoluzzi A, Furini F, Scirè CA (2018). Osteoarthritis and its management-epidemiology, nutritional aspects and environmental factors. Autoimmun Rev.

[23] Ji FJ, Wang LX, Yang HS, Hu A, Yin YL (2019). Review: The roles and functions of glutamine on intestinal health and performance of weaning pigs. Animal.

[24] Farag MR, Alagawany M, El-Hack MEA (2017). Role of chromium in poultry nutrition and health: Beneficial applications and toxic effects. Int J Pharmacol.

[25] Kim JH, Lee HK, Yang TS, Kang HK, Kil DY (2019). Effect of different sources and inclusion levels of dietary fat on productive performance and egg quality in laying hens raised under hot environmental conditions. Asian-Australas J Anim Sci.

[26] Metzler-Zebeli BU, Eklund M, Mosenthin R (2009). Impact of osmoregulatory and methyl donor functions of betaine on intestinal health and performance in poultry. Worlds Poult Sci J.

[27] Kang S, Kim D, Lee S (2020). An acute, rather than progressive, increase in temperature-humidity index has severe effects on mortality in laying hens. Front Vet Sci.

[28] Barnes DM, Song Z, Klasing KC, Bottje W (2002). Protein metabolism during an acute phase response in chickens. Amino Acids.

[29] Lara LJ, Rostagno MH (2013). Impact of heat stress on poultry production. Animals.

[30] Dubrez L, Causse S, Borges Bonan N, Dumétier B, Garrido C (2020). Heat-shock proteins: Chaperoning DNA repair. Oncogene.

[31] Bochman ML, Schwacha A (2008). The Mcm2–7 complex has in vitro helicase activity. Mol Cell.

[32] Mori Y, Inoue Y, Taniyama Y, Tanaka S, Terada Y (2015). Phosphorylation of the centrosomal protein, Cep169, by Cdk1 promotes its dissociation from centrosomes in mitosis. Biochem Biophys Res Commun.

[33] Li J, Poolman RA, Brooks G (1998). Role of G1 phase cyclins and cyclin-dependent kinases during cardiomyocyte hypertrophic growth in rats. Am J Physiol Heart Circ Physiol.

[34] Zhang Y, Morar M, Ealick SE (2008). Structural biology of the purine biosynthetic pathway. Cell Mol Life Sci.

[35] Hille R, Nishino T (1995). Xanthine oxidase and xanthine dehydrogenase. FASEB J.

[36] Venkatachalam KV (2003). Human 3′-phosphoadenosine 5′-phosphosulfate (PAPS) synthase: Biochemistry, molecular biology and genetic deficiency. IUBMB Life.

[37] Conti M, Beavo J (2007). Biochemistry and physiology of cyclic nucleotide phosphodiesterases: Essential components in cyclic nucleotide signaling. Annu Rev Biochem.

[38] Traut TW (1994). Physiological concentrations of purines and pyrimidines. Mol Cell Biochem.

[39] Tang Z, Ye W, Chen H (2019). Role of purines in regulation of metabolic reprogramming. Purinergic Signal.

[40] Nedergaard S, Bolam JP, Greenfield SA (1988). Facilitation of a dendritic calcium conductance by 5-hydroxytryptamine in the substantia nigra. Nature.

[41] Calder PC (2018). Very long-chain n-3 fatty acids and human health: Fact, fiction and the future. Proc Nutr Soc.

[42] Funk CD (2001). Prostaglandins and leukotrienes: Advances in eicosanoid biology. Science.

[43] Liu L, Liu X, Cui H, Liu R, Zhao G, Wen J (2019). Transcriptional insights into key genes and pathways controlling muscle lipid metabolism in broiler chickens. BMC Genomics.

[44] McGarry JD, Brown NF (1997). The mitochondrial carnitine palmitoyltransferase system—from concept to molecular analysis. Eur J Biochem.

[45] Zhang H, Wang S, Wang Z (2012). A genome-wide scan of selective sweeps in two broiler chicken lines divergently selected for abdominal fat content. BMC Genomics.

[46] Muret K, Désert C, Lagoutte L (2019). Long noncoding RNAs in lipid metabolism: Literature review and conservation analysis across species. BMC Genomics.

[47] Wang G, Kim WK, Cline MA, Gilbert ER (2017). Factors affecting adipose tissue development in chickens: A review. Poult Sci.

[48] Saunderson CL, Mackinlay J (1990). Changes in body-weight, composition and hepatic enzyme activities in response to dietary methionine, betaine and choline levels in growing chicks. Br J Nutr.

[49] Zhan Y, Su H, An W (2016). Glycosyltransferases and non-alcoholic fatty liver disease. World J Gastroenterol.

[50] D’Andre HC, Paul W, Shen X (2013). Identification and characterization of genes that control fat deposition in chickens. J Anim Sci Biotechnol.

[51] Lee M, Park H, Heo JM, Choi HJ, Seo S (2021). Multi-tissue transcriptomic analysis reveals that L-methionine supplementation maintains the physiological homeostasis of broiler chickens than D-methionine under acute heat stress. Plos One.

[52] Zhang L, Li P, Liu R (2015). The identification of loci for immune traits in chickens using a genome-wide association study. PloS One.

[53] Baggiolini M (2001). Chemokines in pathology and medicine. J Intern Med.

[54] Alam MS, Costales MG, Cavanaugh C, Williams K (2015). Extracellular adenosine generation in the regulation of pro-inflammatory responses and pathogen colonization. Biomolecules.

[55] Tapinos NI, Polihronis M, Thyphronitis G, Moutsopoulos HM (2002). Characterization of the cysteine-rich secretory protein 3 gene as an early-transcribed gene with a putative role in the pathophysiology of Sjögren’s syndrome. Arthritis Rheum.

[56] Berndt A, Pieper J, Methner U (2006). Circulating γδ T cells in response to *Salmonellaenterica* serovar Enteritidis exposure in chickens. Infect Immun.

