# Supplementary material for: Effect of dietary betaine supplementation on the liver transcriptome profile in broiler chickens under heat stress conditions
Source: Anim Biosci. 2023 Aug 30;36(11):1632–46. doi: 10.5713/ab.23.0228 (PMC10623048; doi:10.5713/ab.23.0228)
Supplement: Supplementary file 1 [file ab-23-0228-Supplementary-Table-1.pdf]

Supplementary Table S1. RNA quality score of broiler chickens raised under thermoneutral (TN) or heat stress (HS) conditions.

Treatment[1]	ID	Condition[2]	RIN[3]	rRNA ratio
PC	1	TN	6.8	1.1
PC	2	TN	8.1	1.0
PC	3	TN	7.0	1.0
PC	4	TN	7.0	1.0
PC	5	TN	8.1	2.0
PC	6	TN	7.0	1.2
NC	1	HS	8.3	1.3
NC	2	HS	7.5	1.3
NC	3	HS	7.8	1.6
NC	4	HS	7.3	1.2
NC	5	HS	7.6	1.0
NC	6	HS	8.6	1.1
BT	1	HS	6.7	1.2
BT	2	HS	7.9	1.2
BT	3	HS	7.5	1.1
BT	4	HS	8.8	1.2
BT	5	HS	8.2	1.2
BT	6	HS	7.7	1.1
Average			7.7	1.2

[1]PC, positive control; NC, negative control; basal diet + 0.2% betaine.

[2]TN, thermoneutral conditions; HS, heat stress conditions.

[3]RIN, RNA integrity number.
