# Supplementary material for: Effect of dietary betaine supplementation on the liver transcriptome profile in broiler chickens under heat stress conditions
Source: Anim Biosci. 2023 Aug 30;36(11):1632–46. doi: 10.5713/ab.23.0228 (PMC10623048; doi:10.5713/ab.23.0228)
Supplement: Supplementary file 2 [file ab-23-0228-Supplementary-Table-2.pdf]

Supplementary Table S2. Primers used for quantitative RT-PCR.

Primer name[1]	Primer sequence (5`-3`)[2]	Tm[3] (°C)	Product size (bp)	GenBank accession number
GAPDH	F: GGTGGTGCTAAGCGTGTTAT<br>R: ACCTCTGTCATCTCTCCACA	50.0 - 65.0	142	NM 204305
MCM3	F: GCTACGGATGATCCTGATTT<br>R: ACAGGCTTGATCATTTTTGC	55.2	161	NM_001006421.2
ALOX5	F: ATGAATGCAGATGGTGAGTT<br>R: TGCCATTATGAAAGCTCCTT	53.2	131	XM_046920513.1
XDH	F: [illegible]<br>R: TCTTCTGGAAGGGATCATAC	55.2	157	NM_205127.2
PTGDS	F: ACCAACTATGACGAGTATGC<br>R: CATCTGCCATACACTTGTCC	55.2	199	NM_204259.2
IL4I1	F: GTATCTGGCTAAACACGACT<br>R: AAGGAAGGACAGGTAAAACC	55.2	130	NM_001099351.4
PLIN1	F: GAAGAAGAATCAGCCTTTGC<br>R: GCTCATAGACCTCACACAC	55.2	164	XM_046924873.1
ACACA	F: CTGTGTTACAACAGAACGTG<br>R: CTCCTAGAAGGGCTTTTCAT	55.2	166	NM_205505.2
GCNT2	F: TACATCATCACCATGCACAA<br>R: ACACACCACCATAGACCA	53.2	199	XM_040663909.2

[1]*GAPDH* , glyceraldehyde-3-phosphate; *MCM* , minichromosome maintenance complex component 3; *ALOX5* , arachidonate 5-lipoxygenase; *XDH* , xanthine dehydrogenase; *PTGDS* , prostaglandin D2 synthase 21kDa (brain); *IL4I1* , interleukin 4 induced 1; *PLIN1* , perilipin 1; *ACACA* , acetyl-CoA carboxylase alpha; *GCNT2* , glucosaminyl (N-acetyl) transferase 2.

[2]F, forward; R, reverse.

[3]Tm, melting temperature.
