# Supplementary material for: Effect of dietary betaine supplementation on the liver transcriptome profile in broiler chickens under heat stress conditions
Source: Anim Biosci. 2023 Aug 30;36(11):1632–46. doi: 10.5713/ab.23.0228 (PMC10623048; doi:10.5713/ab.23.0228)
Supplement: Supplementary file 3 [file ab-23-0228-Supplementary-Table-3.pdf]

Supplementary Table S3. Overview of data processing of broiler chickens in current experiment.

Sample name	Raw Data		After Trimmomatic		Trimming rate	Mapping data	
	Read	%GC(F,R)	Read	%GC(F,R)	(%)	Uniquely alignment rate(%)	Overall alignment rate(%)
Bt-1.log	21,520,593	45	21,083,959	45	2.03%	88.64%	96.31%
Bt-2.log	23,174,481	46	22,758,690	46	1.79%	89.44%	96.74%
Bt-3.log	24,132,808	45	23,606,871	44	2.18%	90.59%	96.73%
Bt-4.log	23,041,017	45	22,549,755	45	2.13%	87.38%	94.38%
Bt-5.log	23,404,065	45	22,953,524	45	1.93%	89.37%	96.27%
Bt-6.log	24,177,737	46	23,633,228	46	2.25%	87.99%	95.67%
NC-1.log	21,657,088	45	21,252,245	45	1.87%	90.01%	96.54%
NC-2.log	19,086,693	44	18,686,804	44	2.10%	89.01%	95.98%
NC-3.log	21,909,558	44	21,440,026	44	2.14%	90.78%	97.00%
NC-4.log	22,813,754	45	22,296,112	44	2.27%	90.09%	96.10%
NC-5.log	21,060,158	46	20,676,384	46	1.82%	89.55%	96.63%
NC-6.log	19,853,051	44	19,400,358	44	2.28%	89.36%	96.97%
PC-1.log	24,033,929	44	23,556,083	44	1.99%	88.11%	96.22%
PC-2.log	21,214,679	44	20,723,824	44	2.31%	90.39%	96.59%
PC-3.log	25,407,114	45	24,873,014	45	2.10%	89.96%	96.59%
PC-4.log	22,507,372	44	22,043,072	44	2.06%	91.07%	96.90%
PC-5.log	19,237,429	45	18,887,926	45	1.82%	89.66%	96.62%
PC-6.log	22,857,067	46	22,508,079	45	1.53%	89.83%	96.37%
