# Supplementary material for: Effect of dietary betaine supplementation on the liver transcriptome profile in broiler chickens under heat stress conditions
Source: Anim Biosci. 2023 Aug 30;36(11):1632–46. doi: 10.5713/ab.23.0228 (PMC10623048; doi:10.5713/ab.23.0228)
Supplement: Supplementary file 4 [file ab-23-0228-Supplementary-Fig-1.pdf]

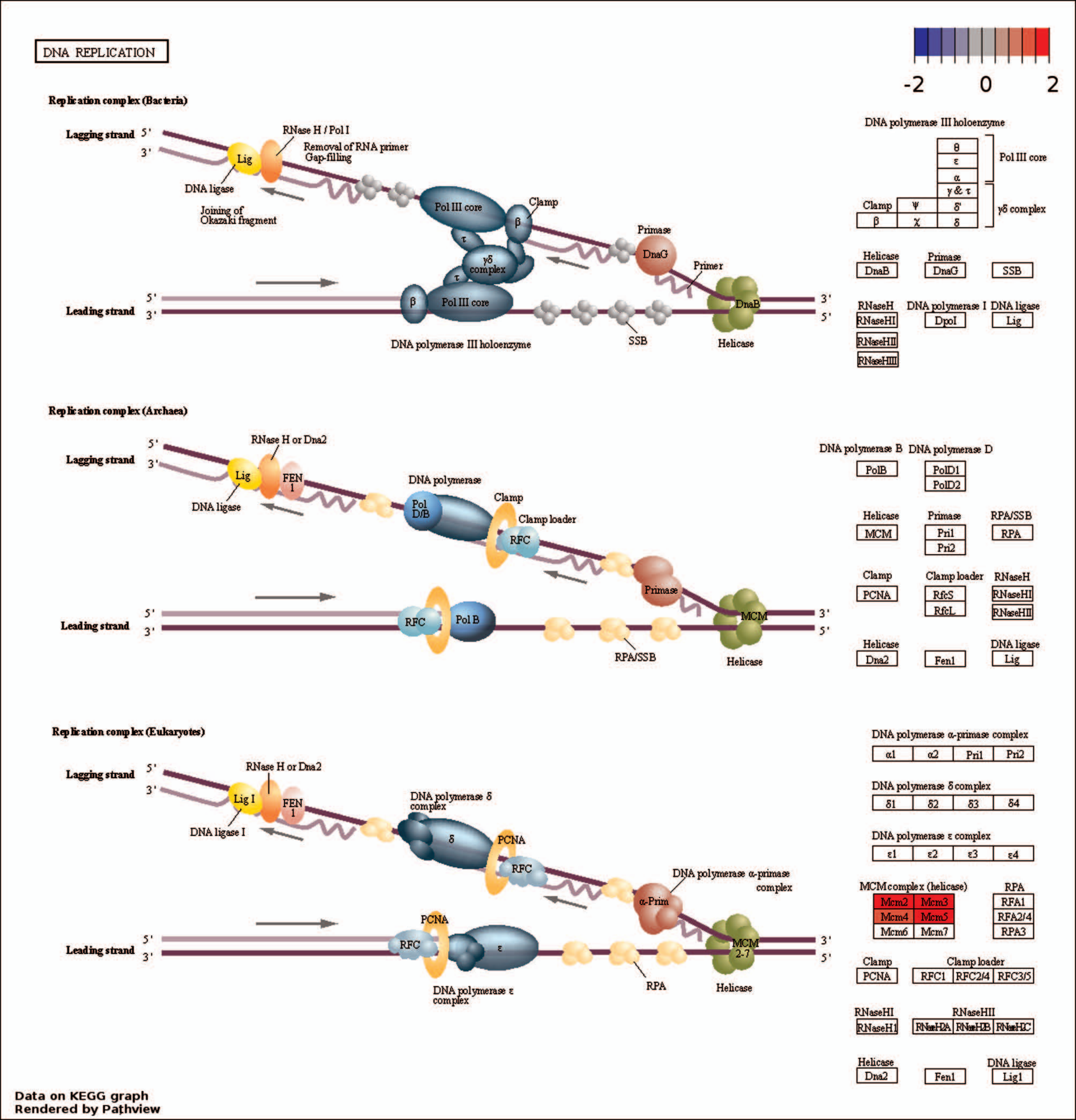

DNA REPLICATION
-2
0
2
Replication complex (Bacteria)
Lagging strand
5'
3'
Lig
RNase H / Pol I
Removal of RNA primer
Gap-filling
DNA ligase
Joining of
Okazaki fragment
Pol III core
Clamp
β
τ
γδ complex
τ
Primase
DnaG
Primer
5'
3'
Leading strand
β
Pol III core
DnaB
3'
5'
DNA polymerase III holoenzyme
SSB
Helicase
DNA polymerase III holoenzyme
θ
ε
α
Pol III core
γ & τ
Clamp
ψ
δ'
γδ complex
β
χ
δ
Helicase
DnaB
Primase
DnaG
SSB
RNaseH
RNaseHI
RNaseHII
RNaseHIII
DNA polymerase I
DpoI
DNA ligase
Lig
Replication complex (Archaea)
Lagging strand
5'
3'
Lig
RNase H or Dna2
FEN 1
DNA ligase
DNA polymerase
Pol D/B
Clamp
Clamp loader
RFC
Primase
MCM
Leading strand
5'
3'
RFC
Pol B
3'
5'
RPA/SSB
Helicase
DNA polymerase B
PolB
DNA polymerase D
PolD1
PolD2
Helicase
MCM
Primase
Pri1
Pri2
RPA/SSB
RPA
Clamp
PCNA
Clamp loader
RfcS
RfcL
RNaseH
RNaseHI
RNaseHII
Helicase
Dna2
Fen1
DNA ligase
Lig
Replication complex (Eukaryotes)
Lagging strand
5'
3'
Lig I
RNase H or Dna2
FEN 1
DNA ligase I
DNA polymerase δ complex
δ
PCNA
RFC
DNA polymerase α-primase complex
α-Prim
Leading strand
5'
3'
PCNA
RFC
ε
MCM 2-7
3'
5'
DNA polymerase ε complex
RPA
Helicase
DNA polymerase α-primase complex
α1
α2
Pri1
Pri2
DNA polymerase δ complex
δ1
δ2
δ3
δ4
DNA polymerase ε complex
ε1
ε2
ε3
ε4
MCM complex (helicase)
Mcm2
Mcm3
Mcm4
Mcm5
Mcm6
Mcm7
RPA
RFA1
RFA2/4
RPA3
Clamp
PCNA
Clamp loader
RFC1
RFC2/4
RFC3/5
RNaseHI
RNaseH1
RNaseHII
RNaseH2A
RNaseH2B
RNaseH2C
Helicase
Dna2
Fen1
DNA ligase
Lig1
Data on KEGG graph
Rendered by Pathview
