# Supplementary figures and images for: Effect of dietary betaine supplementation on the liver transcriptome profile in broiler chickens under heat stress conditions
Source: Anim Biosci. 2023 Aug 30;36(11):1632–46. doi: 10.5713/ab.23.0228 (PMC10623048; doi:10.5713/ab.23.0228)

## TRYPTOPHAN METABOLISM

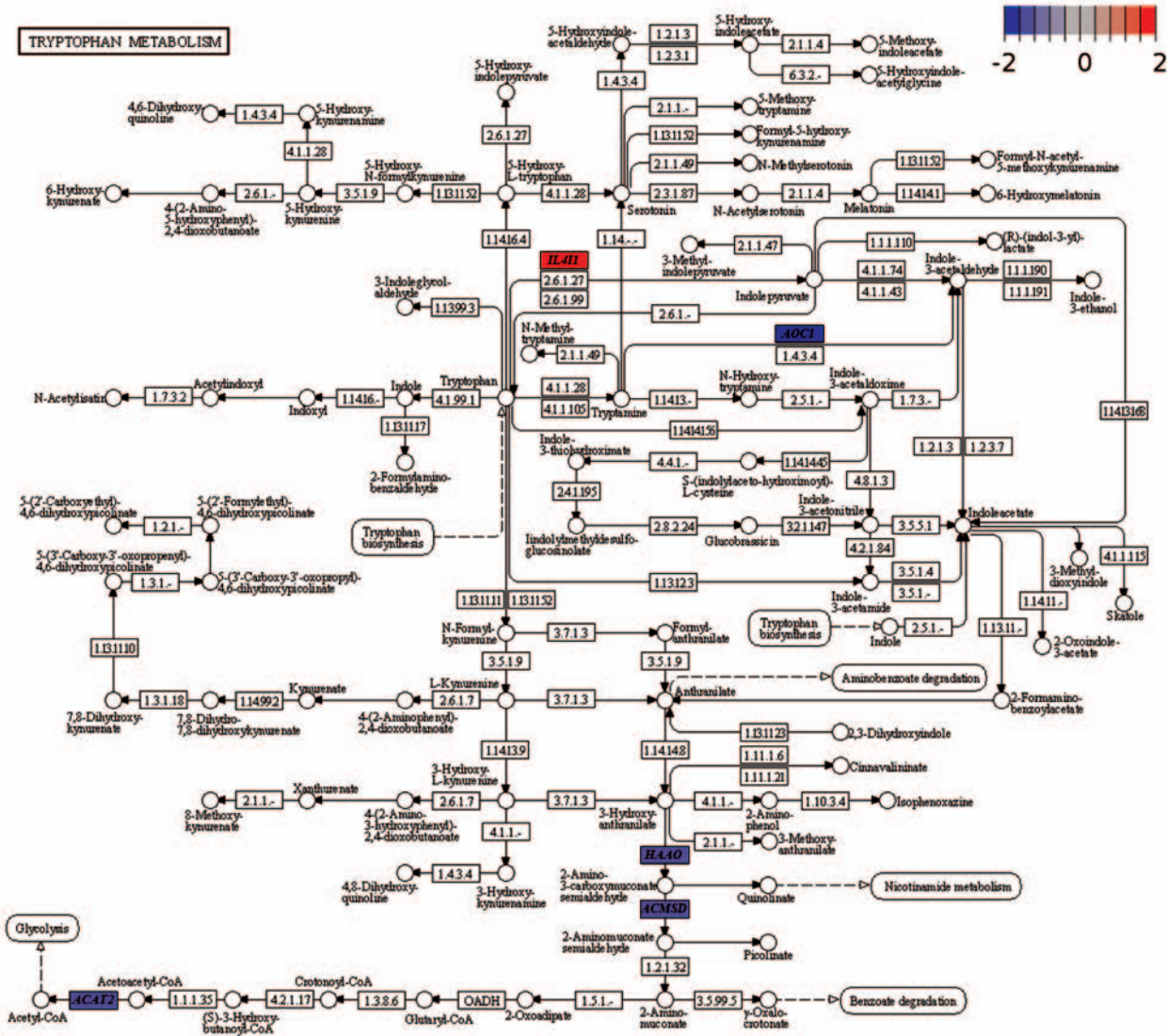

**Data on KEGG graph**  
**Rendered by Pathview**

Supplement: Supplementary file 5 [file ab-23-0228-Supplementary-Fig-2.pdf]

# ARACHIDONIC ACID METABOLISM

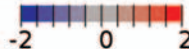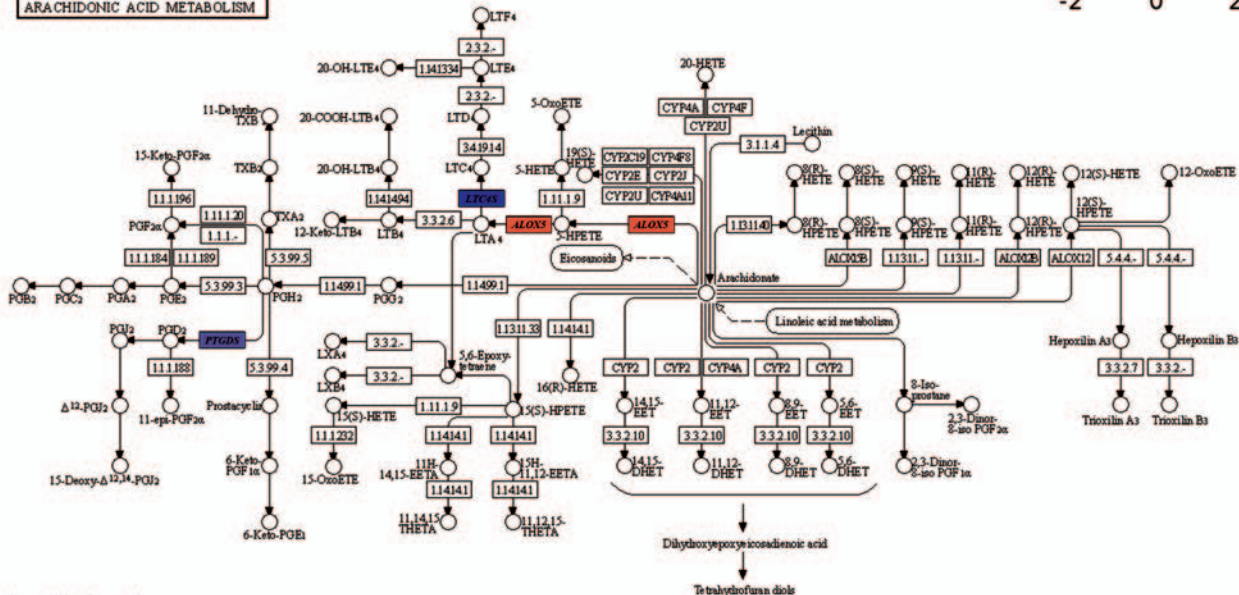

Supplement: Supplementary file 6 [file ab-23-0228-Supplementary-Fig-3.pdf]

# PYRUVATE METABOLISM

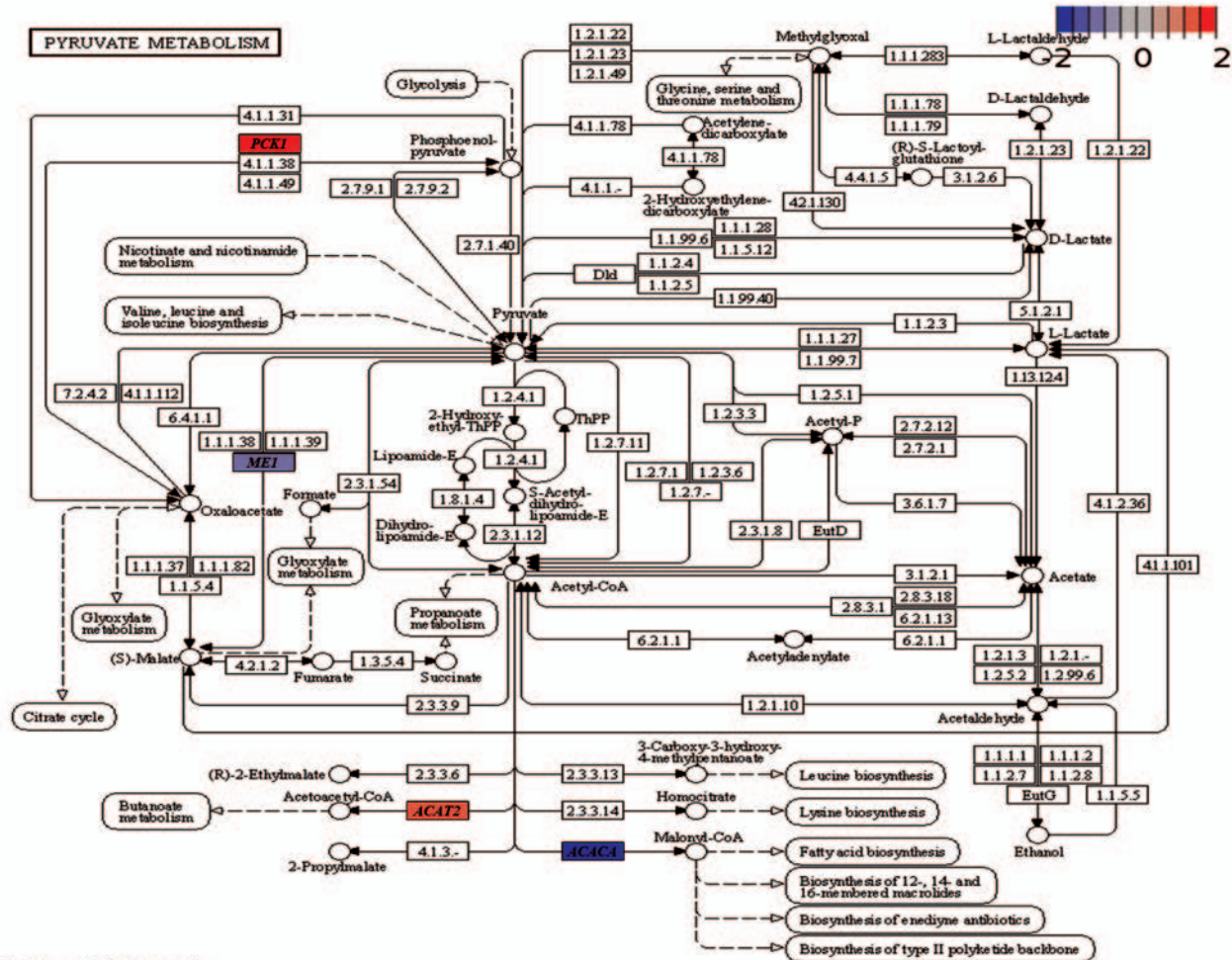

Supplement: Supplementary file 7 [file ab-23-0228-Supplementary-Fig-4.pdf]

# TYROSINE METABOLISM

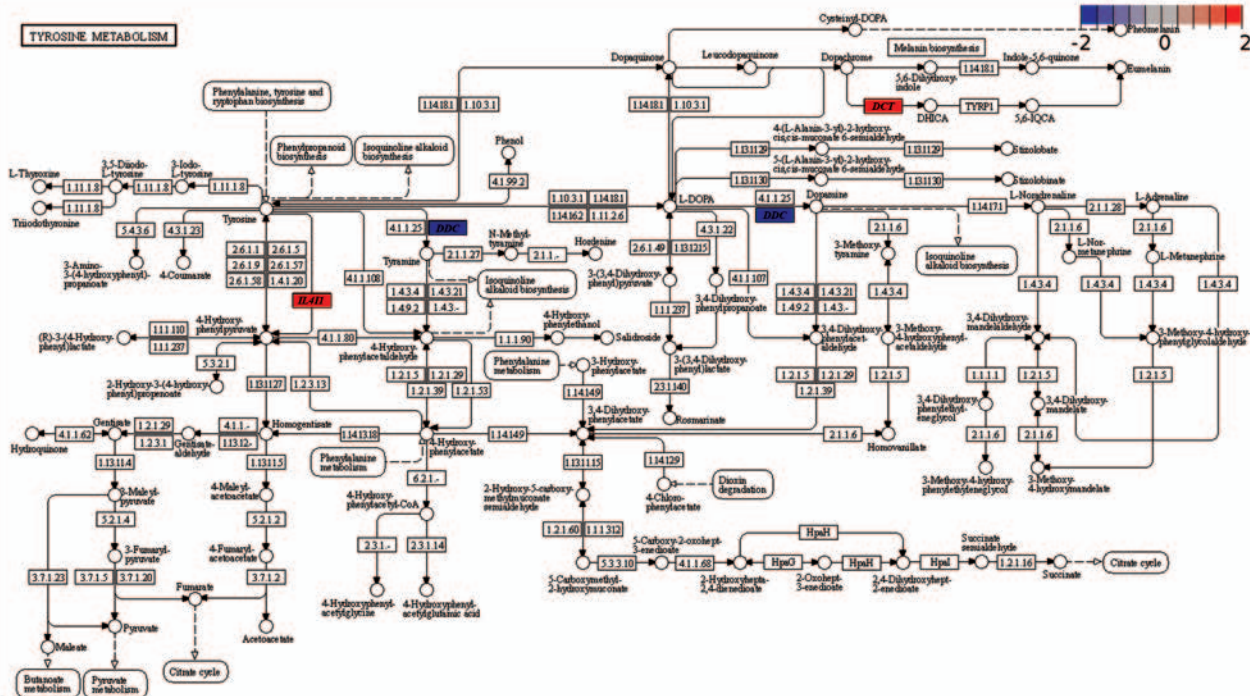

Supplement: Supplementary file 8 [file ab-23-0228-Supplementary-Fig-5.pdf]

## TRYPTOPHAN METABOLISM

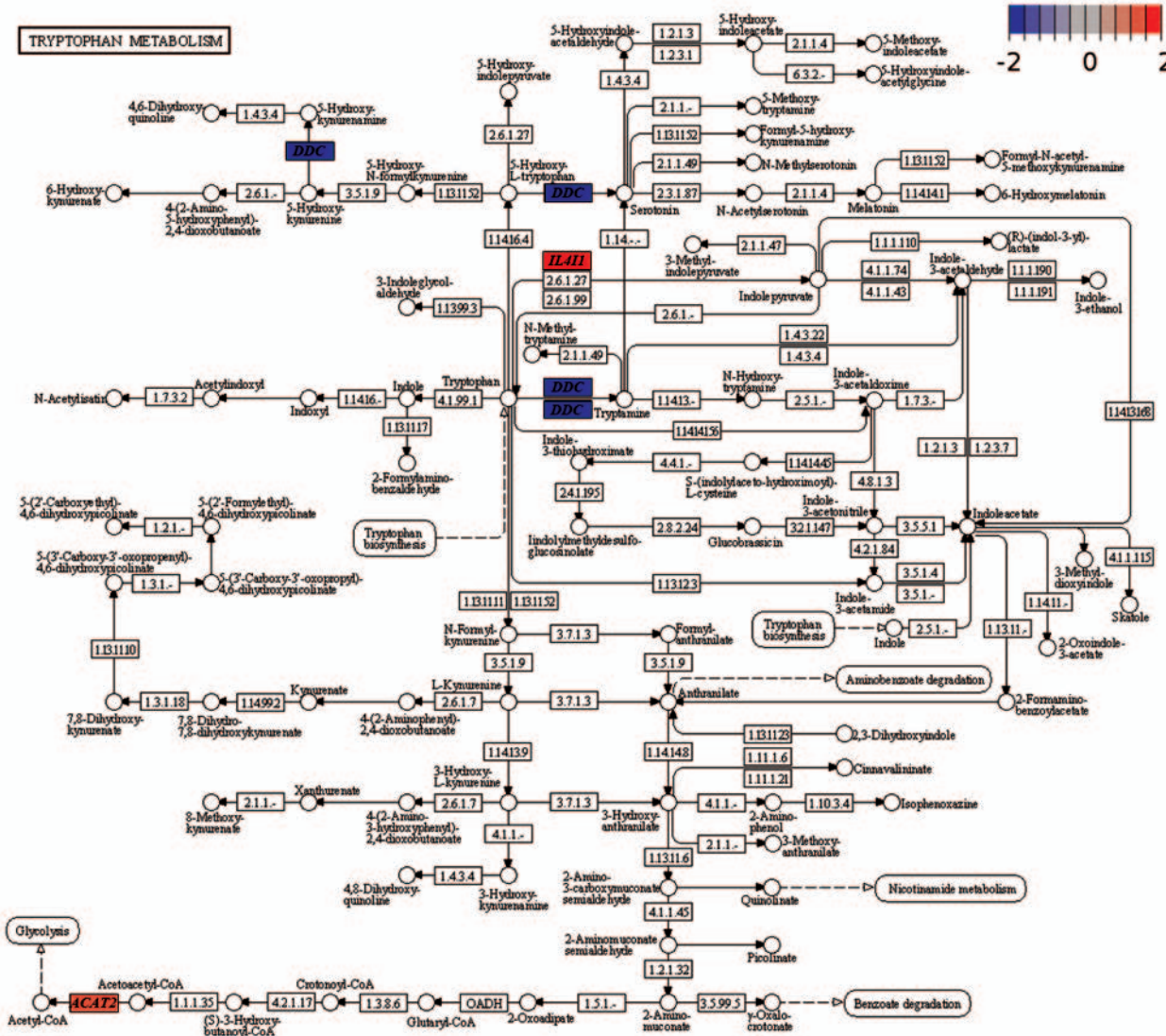

Data on KEGG graph  
Rendered by Pathview

Supplement: Supplementary file 9 [file ab-23-0228-Supplementary-Fig-6.pdf]
